# Supplementary material for: Effects of unilateral neck muscle vibration on standing postural orientation and spatial perception in healthy subjects based on stimulus duration and simultaneous stimulation of trunk muscles
Source: PLoS One. 2023 Jan 26;18(1):e0281012. doi: 10.1371/journal.pone.0281012 (PMC9879387; doi:10.1371/journal.pone.0281012)
Supplement: Attachment — Submitted filename: Response_to_Reviewers.docx [file pone.0281012.s001.docx]

Response to Reviewers

Reviewer #1:

1 In L170, the study subjects are listed as 24 subjects, but since this is a crossover study, the actual number of subjects should be 12.

L148: We tested four conditions: (1) no vibratory stimulation (control), (2) vibratory stimulation of the left neck for 30 s, (3) vibratory stimulation of the left neck for 10 min, and (4) vibratory stimulation of the left neck and left lumbar back for 10 min. Stimulation was performed with the eyes closed while in a resting, sitting position. Measurements in the four conditions were performed with random cross-over using a random number table. Thus, one participant performed a random crossover of the four conditions. In other words, 24 participants performed a total of 96 trials. To facilitate understanding, we have added the relevant sentences. (Line 175)

2 It does not state why the study was limited to males in their 20s as subjects. In the discussion, there is no mention of age- and sex-related differences in balance ability among adolescents. A prior paper, Age and sex differences in human balance performance from 6-18 years of age: A systematic review and meta-analysis. PLoS One. 2019 Apr 9;14(4):e 0214434. shown, and the entire experimental design should be restructured. Regarding postural control, we believe that brain development should also be considered.

L170: The study included 24 participants (all right-handed; 12 males; 25.7±3.7 years; WBL with closed eyes 50.2±2.3%; WBL with open eyes: 50.4±2.8%), and they performed all the four conditions. Thus, the 24 participants included 12 males and 12 females.

L104: the following criteria were established to exclude a possible reduced response to NMV: (1) healthy participants under 65 years of age [20,21], (2) no pain at the site of vibration stimulation [22], (3) no history of the spinal orthopedic disease [23], and (4) no history of the vestibular disease [9]. Therefore, participants over 65 years of age were excluded since aging could have reduced the effectiveness of NMV. On the other hand, as you pointed out, it was not certain from the results of this study whether the same results would be observed in minors or participants aged between 30 and 65 years, who were not included in this study. In addition, this study was not designed to compare sex differences and did not have a sufficiently large sample size to compare the effects of NMV on gender differences. Based on the abovementioned points, we have added a Limitation to the manuscript. (Line 262-264)

Reviewer #2:

Line 22-24: The sentence beginning with “However, the modulating effects….” is difficult to follow. I am unclear on what exactly is being modulated as well as what exactly is unknown – please rephrase this sentence to be clearer (or perhaps separate into two, simpler sentences).

Thank you for pointing out the incomprehensible sentences. We have revised the sentence as follows: “The effects of NMV in healthy participants would vary based on the influence of stimulus duration and combination with trunk muscle vibration.”(Line 22-24 )

Line 48: The use of “almost selectively” here is unclear. Do you mean that focal muscle vibration is not the only activator of primary nerve endings? Or that focal muscle vibration mostly activates primary nerve endings, but also affects other structures? Please clarify.

Thank you for your valuable comments. The previous studies indicated that besides primary nerve endings (Ia), muscle spindle secondary endings (II fibers) and Golgi tendon organs (Ib fibers) could activate slightly. Therefore, we added the word "almost" to the manuscript. However, since it is clear that vibratory stimulation selectively activates the primary nerve endings, we omitted the word "almost."(Line 47)

Line 62: Please change “a previous study” to “previous studies” as you then cite two studies.

Thank you for your valuable comments. We have revised the manuscript accordingly. (Line 61)

Line 75: This sentence was a little difficult to follow; I would recommend rephrasing to clarify that SSA deviates towards the stimulus side during and after NMV, in both healthy and stroke populations (if this is indeed the correct reading). As it is currently, it can be interpreted as “those having a stroke during and after NMV” (as in, those who suffered a stroke during vibration).

Thank you for your valuable suggestion. We have revised the text in the manuscript according to your suggestion as follows: "SSA deviates towards the stimulus side during and after NMV, in both healthy and stroke populations." (Line 74)

Line 82: I believe this sentence is explaining that vibratory stimulus of the neck and lower limbs leads to CoP deflection to the opposite side of stimulus, and vibration of the neck and trunk leads the CoP to the same side as the stimulus. Otherwise, if it is specifying the direction of CoP deflection from those sites individually, the neck would be responsible for both ipsilateral and contralateral deflection. If the first option is true, please make the sentence more explicit about this; if the second option is true, please explain the details of neck vibration causing CoP deflection in both directions.

Thank you for your valuable suggestion. The first option was true. To make the sentence clearer, the description of the lower limb has been omitted. We revised the text in the manuscript as follows: “The local vibratory stimulation of the neck and trunk deviate the standing COP to the same side as the stimulus.” (Line 81-82)

Line 84: The authors state “the direction of standing CoP deflection is similar,” but it is not clear what this direction is similar to. Please clarify.

Thank you for your valuable suggestion. To clarify the sentences, we revised the text in the manuscript as follows: “Moreover, among the stimulated sites of the whole body, when the muscles of the upper trapezius and trunk's lumbar region were vibrated, the standing COP deviated to a similar direction.” (Line 82-84)

Line 107: This is minor, but please remove “the”, as you are referencing any vestibular disease, rather than a particular one.

Thank you for your valuable comments. We have revised the manuscript accordingly. (Line 107)

Line 111: I very much appreciate the inclusion of the frequency and amplitude parameters of vibration, though it would be helpful to readers to include a rationale for these choices (particularly for the amplitude).

Thank you for your valuable suggestion. We have added the citations to the manuscript.

Line 112: The authors reference “anatomical positions” and cite work by Courtine et al; however, this paper makes no explicit reference to any anatomical positions. Please clarify.

Thank you for your valuable suggestion. In some previous studies, the stimulation site of the NMV varied, with some methods fine-tuning the stimulation site by the participant prior to stimulation. In contrast to these previous studies, we used the term “anatomical positions” because we used the same location as the stimulation site for all the participants. However, as you pointed out, the same term was not used in a previous study by Courtine et al. Therefore, we revised the text in the manuscript as follows: “The stimulation terminals were fixed with surgical tape and elastic lumbar bands to the upper trapezius muscle (10 cm medial to the acromion) on the left neck [6, 12] and the multifidus muscle (3 cm lateral to the third lumbar spinous process) on the left lumbar back [6] (Fig 2).” by removing the expression anatomical positions. (Line 111-114)

Line 129: Participants, upon opening their eyes, gazed at “landmarks” – does this refer to the marker placed at eye level on line 126?

Thank you for your valuable suggestion. To improve clarity in the sentences, we revised the text in the manuscript as follows: “Standing COP measurements were performed for 30 s each, starting with closed eyes and then with open eyes while gazing at landmarks placed at eye level 2 m away from the participant.” (Line 126-128)

Line 131: I was unclear on what ‘CoP length’ was referring to. On line 174, the authors refer to “total trajectory length,” which implies – if this refers to the same measure – that it could be pathlength. However, this was not clear from the methods. Either a short description, or a citation would help readers to follow this metric.

Thank you for your valuable suggestion. "COP length" and "COP trajectory length" have been unified to “COP pathlength.”　 In addition, short descriptions have been added as follows: “The pathlength of COP represents the total trajectory followed by the COP from its initial position to its final position. The surface area of COP represents a rectangle bounded by the maximum width of the sway in the ML and AP directions.” (Line 131-133)

Line 138: I am unclear what “sternal pattern” is referring to in this sentence. Was the touch panel projecting an image of their body that they reached for? Or is this just referring to the sternum of each participant?

Thank you for your valuable comments. To understand the position of the participant's right index, we revised the terms to "manubrium of sternum" instead of "sternal pattern."

Participants projected the imaging position of their manubrium of the sternum using their index finger on a touch panel. We revised the text in the manuscript as follows “Participants were instructed to place their right index finger at the position of the manubrium of sternum in a closed eyes state. Subsequently, the participants were instructed to touch the location on the touch panel with their right finger index, which they thought was directly in front of the manubrium of sternum.” (Line 138-142)

Line 140: The participants were given two practice sessions; were they provided any feedback on their practice (or any other) attempts?

Thank you for your valuable comments. We did not provide feedback after two practice sessions. Thus, we added the following sentence: " During the practice sessions, we checked only the procedures and gave no feedback on the results.” (Line 144-145)

Line 147: Were participants allowed to open their eyes to stand? Or did their eyes remain closed the entire time? Similarly, when did data collection start for the eyes open and closed periods?

Thank you for your valuable comments. Standing up was performed with open eyes for safety reasons. After standing up, measurements of standing COP were taken with the eyes closed, then with open eyes starting from the measurer’s instructions. Thus, we revised the text in the manuscript as follows: “Next, the participants stood up with their eyes open, and standing COP was measured with eyes closed for 30 s, and then with open eyes for 30 s.” （Line 150-151）

Line 206: Minor grammar - please change “was no significant difference” to “were no significant differences”

Thank you for your valuable comments. We have revised the manuscript accordingly. (Line 208)

Line 214: “The integration of proprioceptive sensations by NMV” implies that NMV is actually the entity/thing performing the integration. Do the authors mean the influence of NMV on proprioception, perhaps? Please clarify.

Line 215: It is not clear what is meant by “implicated.” Please clarify.

Line 218: The authors state that focal sensory input from NMV alters body representations by modulating the effects of the central nervous system. The preceding lines imply that this may be from NMV exerting some influence on Ia afferents; however, this is never explicitly stated. Please consider a linking sentence to clarify both what “effects of the CNS” represent as well as how - specifically - focal vibration is modulating them.

Thank you for your valuable suggestion. We meant to describe how NMV implicates proprioceptive sensation. We added the effects of NMV on Ia afferents and　added information on the effects of the CNS. However, the specific effect on the CNS is unclear in previous studies as well as from the results of this study. Therefore, we have also added that the specific effects are still unclear. Thus, we revised the text in the manuscript as “Continuing focal sensory input to Ia nerve fibers in muscle spindles induces the illusion of the vibrated muscles being stretched even though they are not actually moving [5]. NMV induces the illusion that the cervical muscles are stretched, indicating that the standing position is tilted. The standing COP bias is considered as the response of CNS to this illusion and is the result of a modulation in the integration of the proprioceptive sensations [30]. The proprioceptive sensations transmitted by type Ia nerve fibers in muscle spindles rapidly communicate the changes in muscle length to the CNS. The broad hierarchy of the CNS composed of the vestibular nucleus [31], cerebellar nucleus ventricle [32], motor cortex [33], temporoparietal junction [34], subcortex [35], and cortex [36] has been implicated in the integration of the proprioceptive sensations. However, the mechanism of effect of NMV on the CNS is still unclear [9,10].” (Line 214-224)

Line 226: The authors state that NMV-induced nervous system modulation is amplified by proprioceptive sensory input to the trunk; however, in this study, vibration of the neck alone did not actually have any significant effects. Is this amplification based on previous literature (in which case it would help to state such), or is the nature of the change less amplification and more attributable to the combination?

Thank you for your valuable suggestion. As you accurately pointed out, vibration of the neck alone did not actually have any significant effects in this study. Previous studies are consistent in reporting that COP is deviated by NMV in amputees and patients with stroke. On the other hand, previous studies on healthy individuals have not reached a definite conclusion on COP bias. Although significant deviation was absent, the results of this study demonstrated that COP tended to gradually deviate to the left side at 30 s NMV and then at 10 min NMV, compared to the control condition, and this difference was significant owing to the combination of vibration stimulation to the trunk muscles. Therefore, we consider that the directionality of COP deviation was amplified by the proprioceptive sensory input to the trunk muscles. However, it is not clear whether this effect is owing to amplification or combination. Based on the abovementioned points, the manuscript text was revised as follows: “This suggests that the NMV-induced nervous system modulation would be amplified by proprioceptive sensory input to the trunk muscles.” (Line 231)

Line 230: It is not clear what is meant by “anterior forehead” and what “component” is being referred to. Please clarify.

Thank you for your valuable suggestion. We revised the text in the manuscript to clarify as follows: “Vibratory stimulation of the upper trapezius muscle and the lumbar region of the trunk, which were the stimulation sites in this study, causes a bias in the standing COP, not only to leftward deviation on the mediolateral plane but also to anterior deviation on the anteroposterior plane [6].” (Line233-236)

Line 241: It would benefit a reader to clarify that this is in reference to a different study, and not the current one.

Thank you for your valuable comments. We have added the relevant text to clarify that these are different studies.

Line 257: This reflects my comments on lines 218 and 226. Please address those comments and update here as appropriate.

Thank you for your valuable suggestion. Kindly refer to the responses concerning the questions regarding lines 218 and 226.
